# Precise pattern of recombination in serotonergic and hypothalamic neurons in a Pdx1-cre transgenic mouse line

**DOI:** 10.1186/1423-0127-17-82

**Published:** 2010-10-17

**Authors:** Gerard Honig, Angela Liou, Miles Berger, Michael S German, Laurence H Tecott

**Affiliations:** 1 Neuroscience Graduate Program, University of California San Francisco, San Francisco, CA, USA; 2 Department of Biochemistry and Molecular Cell Biology, University of California Berkeley, Berkeley, CA, USA; 3 Biomedical Sciences Graduate Program, University of California San Francisco, San Francisco, CA, USA; 4 Diabetes Center, University of California San Francisco, San Francisco, CA, USA; 5 Department of Psychiatry and Center for Neurobiology and Psychiatry, University of California San Francisco, San Francisco, CA, USA; 6 Department of Anesthesiology, Duke University School of Medicine, NC, USA; 7 Molecular Pathogenesis Program & Howard Hughes Medical Institute, Kimmel Center for Biology and Medicine at the Skirball Institute, New York University School of Medicine, New York, USA

## Abstract

**Background:**

Multicellular organisms are characterized by a remarkable diversity of morphologically distinct and functionally specialized cell types. Transgenic techniques for the manipulation of gene expression in specific cellular populations are highly useful for elucidating the development and function of these cellular populations. Given notable similarities in developmental gene expression between pancreatic β-cells and serotonergic neurons, we examined the pattern of Cre-mediated recombination in the nervous system of a widely used mouse line, Pdx1-cre (formal designation, Tg(Ipf1-cre)89.1Dam), in which the expression of Cre recombinase is driven by regulatory elements upstream of the *pdx1 *(pancreatic-duodenal homeobox 1) gene.

**Methods:**

Single (hemizygous) transgenic mice of the *pdx1-cre*^Cre/0 ^genotype were bred to single (hemizygous) transgenic reporter mice (Z/EG and rosa26R lines). Recombination pattern was examined in offspring using whole-mount and sectioned histological preparations at e9.5, e10.5, e11.5, e16.5 and adult developmental stages.

**Results:**

In addition to the previously reported pancreatic recombination, recombination in the developing nervous system and inner ear formation was observed. In the central nervous system, we observed a highly specific pattern of recombination in neuronal progenitors in the ventral brainstem and diencephalon. In the rostral brainstem (r1-r2), recombination occurred in newborn serotonergic neurons. In the caudal brainstem, recombination occurred in non-serotonergic cells. In the adult, this resulted in reporter expression in the vast majority of forebrain-projecting serotonergic neurons (located in the dorsal and median raphe nuclei) but in none of the spinal cord-projecting serotonergic neurons of the caudal raphe nuclei. In the adult caudal brainstem, reporter expression was widespread in the inferior olive nucleus. In the adult hypothalamus, recombination was observed in the arcuate nucleus and dorsomedial hypothalamus. Recombination was not observed in any other region of the central nervous system. Neuronal expression of endogenous *pdx1 *was not observed.

**Conclusions:**

The Pdx1-cre mouse line, and the regulatory elements contained in the corresponding transgene, could be a valuable tool for targeted genetic manipulation of developing forebrain-projecting serotonergic neurons and several other unique neuronal sub-populations. These results suggest that investigators employing this mouse line for studies of pancreatic function should consider the possible contributions of central nervous system effects towards resulting phenotypes.

## Background

The development of methods for the experimental manipulation of gene expression *in vivo *has revolutionized the study of biology. Transgenes which drive expression of recombinases within specific cell types and/or at specific developmental time points are valuable tools for understanding the development and physiology of organ systems *in vivo *[1]. One such system, the mammalian brain, is a remarkably complex and heterogeneous structure comprised of many highly specialized and often rare cell types. Serotonergic neurons, which comprise a tiny fraction of all neurons in the mammalian brain, play an important and unique role in many physiological functions, including the regulation of affect in humans [2]. These neurons are themselves anatomically and functionally diverse, although the molecular, developmental and physiological basis for this diversity is not completely understood [2,3]. Recently, the advent of transgenic methods to express recombinases in all or subsets of serotonergic neurons has provided new insights into the diverse origins and functions of these neurons [4-6].

Serotonergic neurons and pancreatic insulin-producing β-cells exhibit a remarkably similar and specific cascade of transcription factor expression during development, involving the expression of *nkx2.2*, *lmx1b*, and *nkx6.1 *[7,8]. In the pancreas, *pdx1*, a homeodomain transcription factor, plays a critical role in specifying the fate of the early pancreatic primordium and, later in development, is required for successful β-cell development [9]. We hypothesized that regulatory elements which control *pdx1 *expression might be active in the developing brain and might be applied to genetically target serotonergic neurons and/or other neuronal cell types. We therefore examined the developmental pattern of Cre-mediated recombination in the nervous system using a widely used mouse line, Pdx1-cre (formal designation, Tg(Ipf1-cre)89.1Dam) [10-12]. This mouse line has been employed in at least 30 published studies, as it exhibits robust recombination in the developing endocrine pancreas [13,10-41]. Using two Cre reporter lines, Z/EG (Tg(CAG-Bgeo/GFP)21Lbe) and rosa26R (Gt(ROSA)26Sor^tm1Sor^) [42,43,12], we found that that Pdx1-cre also exhibits developmental recombination in the inner ear; in rostral serotonergic neurons; in the hypothalamus; and in non-serotonergic neurons of the caudal hindbrain.

## Materials and methods

### Mice

Strain information is summarized in Table 1. Pdx1-cre and Z/EG mouse lines were maintained as independent colonies of hemizygous transgenic mice; the rosa26R mouse line was maintained as homozygous mutant mice. Strain background was mixed for all lines. Pdx1-cre mice were kindly provided by D. Melton; Z/EG and rosa26R mice were obtained from Jackson Labs. To generate experimental animals, transgenic hemizygous mice from the Pdx1-cre line (genotype *pdx1-cre*^Cre/0^) were bred with hemizygous transgenic mice from the Z/EG line (*Zeg*^GFP/0^) or homozygous transgenic mice from the rosa26R line (*rosa26*^LacZ/LacZ^). Offspring genotypes were obtained in accord with expected Mendelian ratios. Offspring of the following genotypes were used for analysis: *pdx1-cre*^Cre/0^; *Zeg*^GFP/0 ^(experimental), *pdx1-cre*^0/0^; *Zeg*^GFP/0 ^(control); *pdx1-cre*^*Cre*/0^; *rosa26*^LacZ/+ ^(experimental) and *pdx1-cre*^0/0^; *rosa26*^LacZ/+ ^(control). Mice were housed on a 12-hr light-dark cycle in a controlled climate and were fed *ad lib *with Purina LabDiet 5053 mouse chow. All studies involving mice were approved by the UCSF Institutional Animal Care and Use Committee.

**Table 1:** Transgenic mouse lines and relevant genotypes employed

Formal designation	Common designation	Transgene design	Trans-gene insertion	Purpose	WT allele	Trans-genic allele	Geno-types analyzed	Initial reference	MGI ID
Tg(Ipf1-cre)89.1Dam	Pdx1-cre	5.5-kb portion of the mouse *pdx1 *promoter fused to *cre *cassette	Random insertion (pro-nuclear injection	Expression of Cre is driven by regulatory elements which regulate *pdx1 *expression	0	Cre	Cre/0 (hemi-zygote); 0/0 (wild-type control)	*Develop-ment *2002, **129**(10):2447-2457	2684317
Tg(CAG-Bgeo/GFP) 21Lbe	Z/EG	Ubiquitous recombinant promoter, followed by LoxP-flanked *LacZ *cassette, followed by *GFP *cassette	Random insertion with screening for high expression in ES cells (ES cell electro-poration)	When *cre *is expressed in a cell, *LacZ *cassette is excised, leading to *GFP *expression in that cell and all daughter cells	0	GFP	GFP/0 (hemi-zygote)	*Genesis *2000, **28**(3-4):147-155	3046177
Gt(ROSA)26 Sor^**tm1Sor**^	Rosa26R	LoxP-flanked stop cassette followed by *LacZ *cassette	Targeted to the ubiquitously expressing *Gt(ROSA)26Sor *locus	When *cre *is expressed in a cell, stop cassette is excised, leading to *LacZ *expression in that cell and all daughter cells	+	LacZ	LacZ/+ (hetero-zygote)	*Nature Genetics *1999, **21**(1):70-71	1861932

### Genotyping

**Figure 1:**
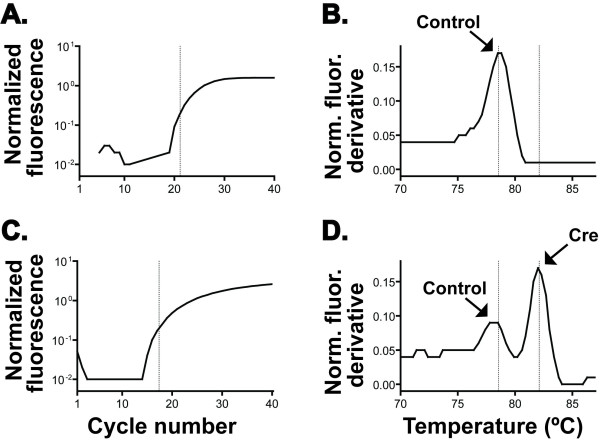
**Transgenic mouse genotyping using multiplex allele-specific PCR and melting curve analysis**. PCR was performed in an optical cycler (ABI 7300) using 1-10 ng genomic DNA from mice of the indicated genotypes and a reagent mix containing SYBR GreenER. Amplification plots (A, C) and melting curves (B, D) are shown. Primers were designed to amplify 2 specific products: a genomic control product, generated from any genomic mouse template; and a transgene-specific product, generated only from genomic templates containing a c*re *transgene. Normalized fluorescence (*y*-axis, A &C) is the baseline-subtracted ratio of SYBR signal to ROX (passive reference dye) signal during amplification cycling (A, C). Normalized fluorescence derivative (*y*-axis, B & D) is the 2^nd ^derivative of normalized fluorescence during the melting curve step. Dotted lines (A, C) indicate cycle threshold. **A & B. **Genomic DNA from a wild-type mouse; note robust amplification of the genomic control product with a single melting peak (allowing the distinction of a negative result from a failed PCR). **C & D. **Genomic DNA from a *pdx1-cre*^Cre/0 ^mouse; note robust amplification with 2 distinct melting peaks corresponding to the control and *cre-*specific products. Arrows indicate presence of the genomic control product (Control) and the transgene-specific product (Cre).

Ear punches or embryonic tails were digested in strip tubes with 0.05 U proteinase K (03115887001, Roche) in 50 μL of DirectPCR Lysis Reagent (402-E, Viagen Biotech) diluted with 50 μL water. 5 μL PCRs were performed using SYBR GreenER PCR mix (Invitrogen), primers (concentration depends on the assay, generally 200 nM) and 0.15 μL of heat-inactivated genomic DNA solution. Thermal cycling was performed on an ABI 7300 instrument with SYBR detection as follows: 95°C for 10 min; 95°C for 15 s followed by 60°C for 1 min (40 cycles); 95°C for 15 s; 60°C for 15 s; followed by a melting curve step with a 2% ramp rate from 60°C to 95°C. Allele-specific PCR products were identified using melting curve analysis as described in Results and Figure 1. Primer sequences and concentrations for assays are provided in Table 2.

**Table 2:** Primer sequences and reaction conditions for gel-free genotyping assays

Assay name	Target locus	Primer 1	Conc. (nM)	Primer 2	Conc. (nM)	Primer 3	Conc. (nM)	Primer 4	Conc. (nM)	Expected results
Z/EG	Any transgene generated using the pCAGG construct (e.g., Z/EG line)	TCGA TGCA GGAT AACTT CGT	400	GGT ACC GTC GACT GCA GAAT	400	AGC AGC AGG CAG GGC TTT	50	GTCT GGA CAC GGG AGC ACTT	50	Primers 3 & 4 generate a single peak in all samples (control product, *gdf*); primers 1 & 2 generate an additional, lower Tm peak (transgene-specific product) in *Zeg*^GFP/0 ^or Zeg^GFP/GFP ^samples.
Cre	Any transgene containing *cre *(e.g., Pdx1-cre line)	ACATT TGGG CCAG CTAAA CAT	200	CGG CATC AAC GTTT TCTT TT	200	GGC GAG AGC AGA GTGT GGA T	200	AAGT CGG CAG GCA CAG GAG	200	Primers 3 & 4 generate a single peak in all samples (control product, *k17*); primers 1 & 2 generate an additional, higher Tm peak (transgene-specific product) in any sample with a *cre *transgene.
PdxCre	*cre *fused to 5' regulatory region of pdx1 gene (Pdx1-cre line)	TAAG GCCT GGCT TGTA GCTC	200	ACC GGT AATG CAG GCA AAT	200	AGC AGC AGG CAG GGC TTT	30	GTCT GGA CAC GGG AGC ACTT	30	Primers 3 & 4 generate a single peak in all samples (control product); primers 1 & 2 generate an additional, lower Tm peak (transgene-specific product) in *pdx1-cre*^Cre/0 ^or *pdx1-cre*^Cre/Cre ^samples.
Rosa26	Any rosa26 allele targeted using a standard targeting allele (e.g., Rosa26R line)	GCGC GCGC GCGT GATC TGCA ACTC CAGT CTTTC	200	GCG CGC GCG CGC GCG CGC GCC ACAC CAG GTTA GCC TTTA AGC	200	GAC AGG ATAA GTAT GAC ATCA TCAA GG	200			Primers 1 & 2 generate a single peak in samples containing the wild-type *rosa26 *allele; primers 2 & 3 generate a lower Tm peak (transgene-specific product) in samples containing a targeted allele; both peaks are observed in heterozygote samples.

### Dissection and histology

4 mice or embryos were analyzed for each genotype and developmental stage. Timed matings were carried out with embryonic day 0.5 considered to be midday of the day of discovery of a vaginal plug. For whole-mount preparations, e10.5 embryos were dissected and briefly fixed in 4% para-formaldehyde in phosphate-buffered saline (PBS), then incubated overnight at 37°C in β-galactosidase (LacZ) staining media (10 mM Tris-HCl pH 7.4, 5 mM K_4_FeCN_6_, 5 mM K_3_FeCN_6_, 2 mM MgCl_2 _and 0.8 mg/ml X-gal (Invitrogen)). For embryonic samples, embryos were dissected at the appropriate stage and immediately embedded and frozen without fixation in Optimal Cutting Temperature media (Tissue-Tek). Embedded embryos were sectioned (transverse, 20 μm) on a cryostat. For adult samples, mice were deeply anesthetized and then perfused with phosphate-buffered saline (PBS) followed by 4% para-formaldehyde in PBS; brains were removed, cryoprotected in 30% sucrose in PBS and sectioned (50 μm, saggital) on a freezing microtome. Immunostaining was performed on-slide for embryonic samples and free-floating for adult tissues. Sections were incubated in blocking solution (4% goat serum, 2% BSA and 0.1% Triton-X-100 in PBS) for 1 h; incubated with primary antibody diluted in blocking solution for 18 h at 4°C; washed in PBS; incubated for 2 h with appropriate secondary anti-IgG antibodies conjugated to Alexa 488 or Alexa 594 dyes; and washed and mounted in Vectashield media (Vector Laboratories). Tyramide signal amplification (TSA) reagents (Invitrogen) were used as per manufacturer's instructions. For β-galactosidase staining of sections, sections were incubated overnight in staining media as described above. Slides were imaged using a confocal, upright epifluorescence or brightfield dissection microscope. Red and green fluorescence spectra were captured separately and appropriate control experiments were performed to confirm specificity and lack of cross-reactivity in labeling.

### Antibodies

The following primary antibodies and dilutions were used: chicken α-GFP, 1:1000 (Aves Labs); rabbit α-serotonin, 1:6000 (Immunostar); mouse α-TPH (tryptophan hydroxylase), 1:200 (Sigma); mouse α-Nkx2.2 (Developmental Studies Hybridoma Bank, clone 74.5A5); mouse α-Mash1, 1:100 (BD Transduction); rabbit α-Pdx1 (generated in lab??), 1:1000; and mouse α-Isl1, 1:50 (Developmental Studies Hybridoma Bank, clone 40.206).

## Results and discussion

As the efficiency of Cre-mediated recombination is not necessarily identical across different target loci, we intercrossed Pdx1-cre transgenic mice with mice from two distinct Cre reporter lines. The transgenes and genotypes analyzed are summarized in Table 1 and in the Methods. In brief, the reporter lines function as follows: the Z/EG line carries a single-copy transgene containing a strong and ubiquitous recombinant promoter, followed by a β-galactosidase and transcriptional stop cassette flanked by loxP sites, followed by a GFP cassette [42]. In Cre-negative cells, β-galactosidase only is expressed; in Cre-expressing lineages, the β-galactosidase cassette is excised, permitting expression of GFP. The Rosa26R mouse line harbors a transgene inserted by targeted mutagenesis into the ubiquitously expressed *rosa26 *locus; this transgene consists of a loxP-flanked stop cassette followed by a β-galactosidase cassette [43]. In Cre-negative cells, no reporter β-galactosidase transgene is expressed; in Cre-expressing lineages, the stop cassette is excised, permitting expression of β-galactosidase.

Standard polymerase chain reaction (PCR) genotyping protocols for the mouse lines used have been described previously [10,42,43]. We adapted previously published methods for single nucleotide polymorphism detection [44-50] to develop a gel-free genotyping method based on multiplex PCR and discrimination of allele-specific products using SYBR-Green-detected melting curve analysis. Small-product multiplex PCR reactions are performed using an optical cycler with the inclusion of SYBR Green dye, which fluoresces in the presence of double-stranded DNA. At the end of the amplification reaction, PCR products corresponding to specific alleles are detected by progressively heating the reaction and plotting the derivative of SYBR fluorescence; annealing and melting of a specific product generates a peak at a specific melting temperature. The PCR reaction well is never opened and no gels are required. Melting curve peaks (position and shape) can be manipulated using simple, inexpensive primer modification [45], such that this approach can readily address most PCR based, multiplex genotyping applications. This method confers significant advantages over existing methods, including higher throughput, uniform and robust PCR conditions, low cost and reduction of post-PCR contamination. Representative data, from an assay used to genotype mice of the Pdx1-cre line, is provided in Figure 1. Detailed instructions for assay design and implementation are available upon request (also see [45,44-50]). Primer sequences and reaction conditions are provided in Table 2.

**Figure 2:**
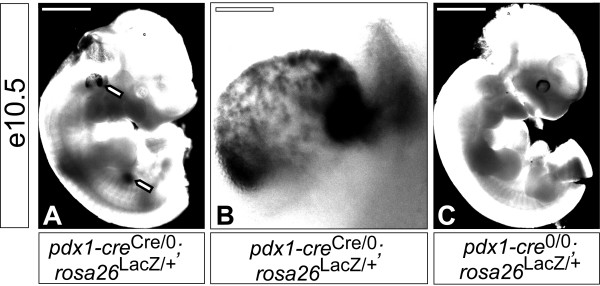
**Cre-mediated recombination in the pancreatic primordium and inner ear in the e10.5 embryo**. Whole-mount images of a *pdx1-cre*^Cre/0^; *rosa26*^LacZ/+ ^embryo (A & B) and a *pdx1-cre*^0/0^; *rosa26*^LacZ/+ ^embryo (C) processed for β-galactosidase activity. β-galactosidase activity was observed in the pancreatic primordium (bottom arrow, left panel) and inner ear formation (top arrow, A; region in higher magnification in B). β-galactosidase activity was not evident in *pdx1-cre*^0/0^; *rosa26*^LacZ/+ ^control embryos (C). Scale bars, 1 mm (A and C) and 150 μm (B).

**Figure 3:**
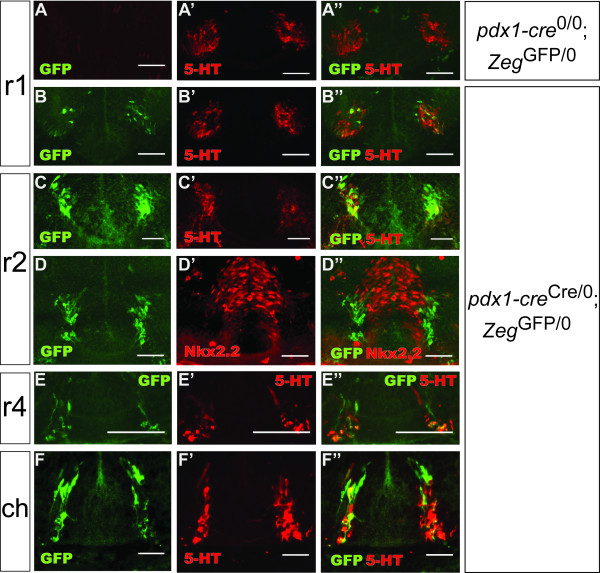
**Cre-mediated recombination coincides with serotonergic neurogenesis in the e11.5 embryo**. Epifluorescence images of the e11.5 developing hindbrain of embryos, transversely sectioned, immunostained for GFP (green) and 5-HT or Nkx2.2 (red). In *pdx1-cre*^Cre/0^; *Zeg*^GFP/0 ^embryos, GFP was always expressed in or adjacent to newborn serotonergic neurons. Both serotonergic and non-serotonergic neurons expressed GFP in the rostral hindbrain (B, C, D) (rhombomere 1, r1; rhombomere 2, r2) and caudal hindbrain (ch) (F). The degree of co-expression of GFP and the serotonergic phenotype was greatest in the rostral hindbrain, with little overlap in the caudal hindbrain and sparse GFP expression in rhombomere 4 (r4) (E). GFP was not expressed in the Nkx2.2^+ ^progenitor zone (D). GFP expression was not evident in sections from a *pdx1-cre*^0/0^; *Zeg*^GFP/0 ^embryo (A). Scale bars, 100 μm.

**Figure 4:**
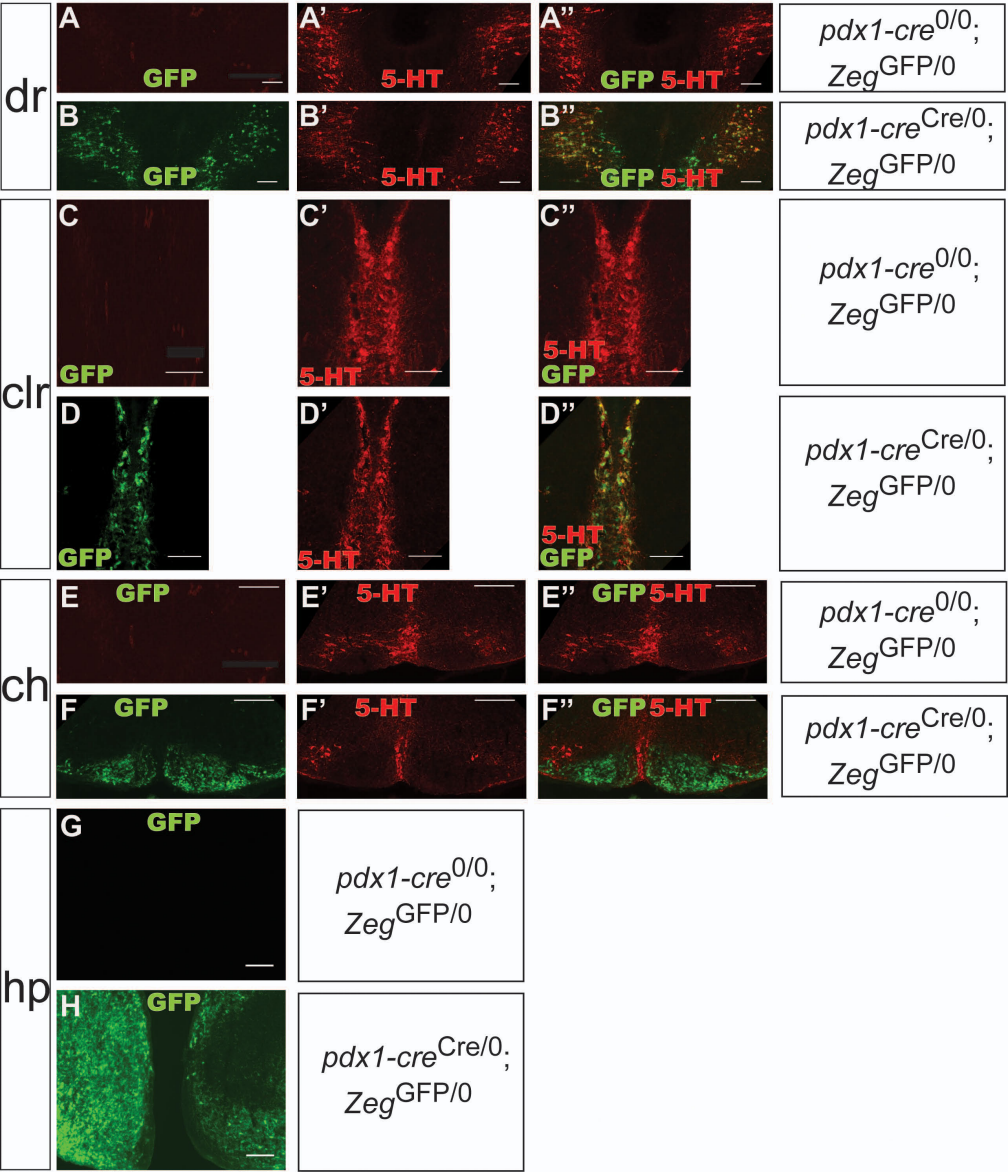
**Cre-mediated recombination in the hindbrain and diencephalon in the e16.5 embryo**. Epifluorescence images from *pdx1-cre*^Cre/0^; *Zeg*^GFP/0 ^(B, D, F, H) and *pdx1-cre*^0/0^; *Zeg*^GFP/0 ^(A, C, E, G) e16.5 embryos, transversely sectioned, immunostained for GFP (green) and 5-HT (red). GFP was expressed in the dorsal raphe nucleus (dr) (B), caudal linear raphe (clr) (D), caudal hindbrain (ch) (F) and hypothalamus (hp) (H). In the rostral hindbrain, GFP expression occurred in the serotonergic dorsal raphe and caudal linear nuclei (B, D). In the caudal hindbrain, GFP expression was observed in the non-serotonergic inferior olive nucleus, adjacent to serotonergic raphe nuclei (F). GFP expression was not evident in sections from *pdx1-cre*^0/0^; *Zeg*^GFP/0 ^control embryos (A, C, E, G). Scale bars, 100 μm.

Under our experimental conditions, reporter expression of GFP (Z/EG line) or LacZ (rosa26R line) provided a specific marker of Cre-mediated recombination. No reporter expression was evident in *pdx1-cre*^0/0^; *Zeg*^GFP/0 ^and *pdx1-cre*^0/0^; *rosa26*^LacZ/+ ^littermate control tissues (Figures 2C; 3A; 4A; C; E; G). GFP and LacZ expression patterns described below were observed in *pdx1-cre*^Cre/0^; *Zeg*^GFP/0 ^and *pdx1-cre*^Cre/0^; *rosa26*^LacZ/+ ^mice or embryos. For each genotype and time point, comparable patterns of GFP or LacZ expression were observed in all analyzed animals (n = 4).

**Figure 5:**
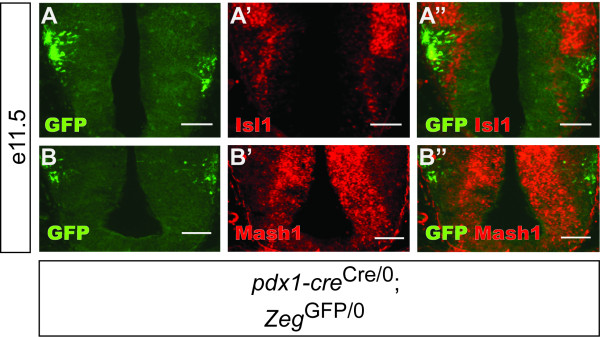
**Cre-mediated recombination in the ventral diencephalon in the e11.5 embryo**. Epifluorescence images of the e11.5 diencephalon of *pdx1-cre*^Cre/0^; *Zeg*^GFP/0 ^embryos, transversely sectioned, immunostained for GFP (green) and Isl1 (A) or Mash1 (B) (red). GFP was not expressed at the ventral surface near the floor plate adjacent to the Isl1^+ ^Mash1^+ ^neurogenic zone. Scale bars, 100 μm.

Recombination was first detected at e10.5 in the pancreas (Figure 2A), as reported [10], and in the inner ear formation (patchy expression in the developing anterior and posterior semicircular canal region with enriched expression in two anterior and posterior medial domains) (Figures 2A &2B). The earliest recombination in the central nervous system was observed at e11.5, in the hindbrain (Figure 3) and in the diencephalon (Figure 5).

In the e11.5 hindbrain, GFP expression was observed to spatiotemporally coincide with serotonergic neurogenesis. In rhombomeres 1 (r1) and 2 (r2), GFP was observed exclusively within a ventral zone where serotonin neurons are first observed in the developing brain[8] (Figures 3B, C, D). In r1 and r2, most GFP^+ ^cells were newborn serotonergic neurons, as identified by serotonin (5-HT) immunoreactivity, although a small minority of GFP-labeled cells appeared to be 5-HT^- ^and some 5-HT^+ ^cells were GFP^-^, suggesting that recombination in this cell lineage was mosaic at this time point (Figures 3B, C, E). GFP expression was observed adjacent to but not in the Nkx2.2^+ ^progenitor zone (Figure 3D), suggesting that Cre is first expressed as serotonergic cells differentiate and migrate out of this progenitor zone. In rhombomere 4 (r4), serotonergic neurons are not generated[8], although 5-HT^+ ^fibers can be detected (distinguished from cell bodies by morphology, anatomical location and lack of DAPI staining); in r4, GFP expression was observed in most 5-HT^+ ^fibers and rare 5-HT^- ^cell bodies (Figure 3E). In the caudal hindbrain, serotonin and reporter immunoreactivity was always observed in the same sections, but rarely within the same cells; cellular GFP immunoreactivity was observed immediately dorsal to most 5-HT^+ ^neurons (Figure 3F). In general, we observed a rostral-caudal gradient of overlap between GFP expression and the serotonergic phenotype in the hindbrain.

**Figure 6:**
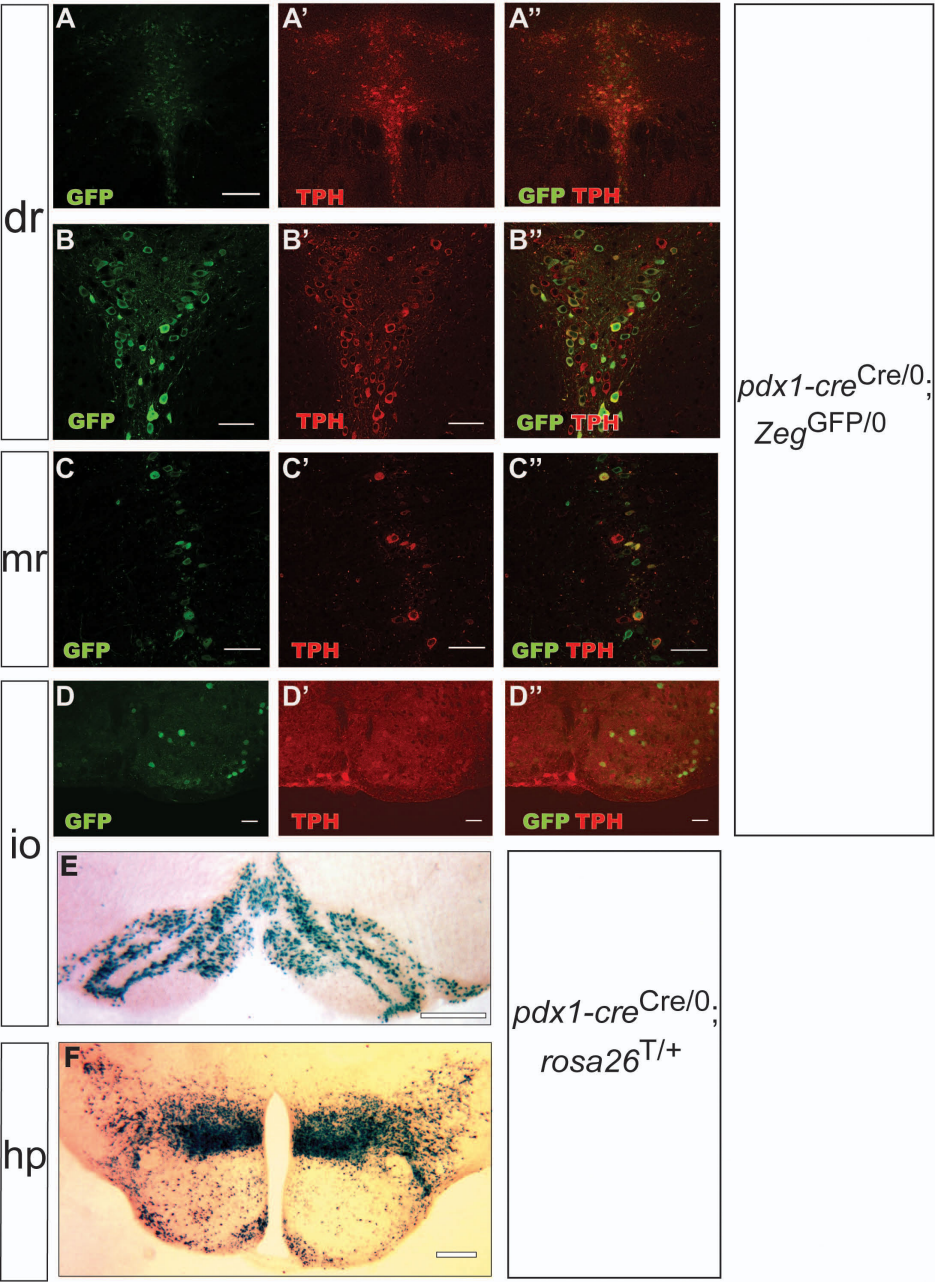
**Cre-mediated recombination in forebrain-projecting serotonergic neurons, inferior olive neurons and hypothalamic neurons in the adult brain**. A-D: Individual optical sections obtained using confocal imaging of saggital sections from adult *pdx1-cre*^Cre/0^; *Zeg*^GFP/0 ^mice. **A**: Wide-field image of the serotonergic dorsal raphe nucleus (dr) demonstrating extensive and anatomically restricted expression of TPH and GFP in this structure. **B**: Higher-magnification image of the dorsal raphe nucleus: a large majority of TPH^+ ^neurons express GFP and that all GFP^+ ^cells in this region are serotonergic neurons. **C**: In the median raphe nucleus (mr), there was partial overlap between GFP and TPH expression. **D**: In the caudal hindbrain, GFP expression was observed in the inferior olive nucleus (io), adjacent to but not overlapping with serotonergic raphe nuclei. **E-F**: Brightfield images of saggital sections obtained from adult *pdx1-cre*^Cre/0^; *rosa26*^LacZ/+ ^mice, processed for LacZ activity. **E**: The inferior olive nucleus was labeled with LacZ. **F**: Multiple nuclei of the hypothalamus, notably the dorsomedial, lateral and arcuate nuclei, were labeled with LacZ. Scale bars: 80 μm (A); 60 μm (B, C, D); 200 μm (E); 150 μm (F).

The recombination pattern observed in the e11.5 hindbrain predicted the pattern we observed at later stages of development. At e16.5, reporter expression in the rostral hindbrain was restricted to the rostral raphe nuclei, particularly the dorsal raphe nucleus (Figure 4B) and caudal linear raphe nucleus (Figure 4D). In the caudal hindbrain, reporter expression was observed in the non-serotonergic inferior olive nucleus but not in adjacent serotonergic neurons (Figure 4F). In adult tissues, confocal microscopy was employed for analysis of hindbrain sections in order to more rigorously analyze the co-expression of GFP and the serotonergic phenotype. In the dorsal raphe nucleus, which is generated in r1, the most rostral portion of the developing hindbrain [4], the large majority of serotonergic neurons (identified by immunoreactivity for tryptophan hydroxylase, or TPH) expressed GFP, and all GFP-positive cells were serotonergic (Figures 6B, B', B''). In the median raphe nucleus, which is generated in r1, r2 and r3 [4], there was partial overlap between GFP and 5-HT expression (Figures 6C, C', C''). In the caudal hindbrain, 5-HT and GFP expression were completely non-overlapping, although occurring in the same sections; recombination was restricted to the inferior olive nucleus (Figures 6D, D', D'', E). Interestingly, the inferior olive can be similarly labeled using a Cre line in which the *cre *was introduced into the locus for *ptf1a*, a transcription factor which interacts with *pdx1 *during early pancreatic development [51,52]. Given the anatomical localization of GFP^+ ^5-HT^+ ^cells in the adult, it is likely that a vast majority of forebrain-projecting serotonergic neurons, and virtually no spinal-cord-projecting serotonergic neurons, exhibit Cre-mediated recombination in the Pdx1-cre line[2]. These data provide further evidence that caudal and rostral serotonergic neurons, though generated through highly similar developmental processes [8], exhibit distinct patterns of gene expression regulation [3]. This Pdx1-cre mouse line may be a useful resource for investigators interested in manipulating gene expression in serotonergic neurons projecting to the forebrain but not to the brainstem and spinal cord.

In the e11.5 diencephalon, recombination occurred, as in the hindbrain, in a restricted ventral zone of the neural tube, adjacent to neurogenic zones (here identified by Isl1 and Mash1 expression) (Figure 5). This developmental pattern resulted in GFP and LacZ expression in specific, anatomically defined nuclei of the hypothalamus, as could be observed at e16.5 (Figure 4H) and especially in adult sections. In the adult hypothalamus, reporter expression occurred in the arcuate nucleus, dorsomedial nucleus and lateral hypothalamus (Figure 6F). These regions of the hypothalamus are all critically involved in the *in vivo *regulation of metabolic functions such as glucose homeostasis [53]. The dorsomedial hypothalamic nucleus is relatively poorly characterized at the molecular level, and to our knowledge no transgenic mouse line has been reported which exhibits specific transgene expression in this sub-region of the hypothalamus. The possible existence of specific pdx1 regulatory sequences directing expression in the dorsomedial hypothalamic nucleus could be used to generate such transgenic mouse lines for the study of this important hypothalamic cell population. Interestingly, many hypothalamic neurons share specialized physiological attributes with β-cells, such as glucose sensing[54], and Cre transgenes generated using the *insulin *and *ptf1a *promoters produce recombination in the hypothalamus [51,55].

**Figure 7:**
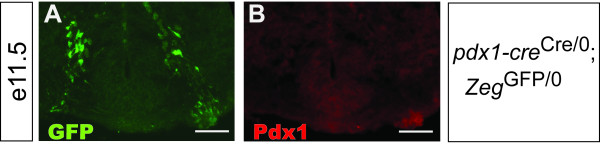
**Lack of detectable expression of endogenous *pdx1 *in the mouse hindbrain**. Section from the r1 region of a *pdx1-cre*^Cre/0^; *Zeg*^GFP/0 ^embryo, immunostained for GFP (A) and Pdx1 (B) using TSA amplification. No detectable expression of Pdx1 was observed, despite robust expression of GFP. Scale bars, 100 μm.

Widespread expression of endogenous *pdx1 *in the rat brain has been reported [56,57]. Our results suggested a more restricted pattern of endogenous *pdx1 *expression might occur in the mouse central nervous system. We therefore attempted to detect expression in the mouse brain of *pdx1 *at various developmental stages using immunofluorescence. We were unable to detect endogenous *pdx1 *expression at any time point, including the earliest time point at which Cre-mediated recombination was observed: tyramide signal amplification of Pdx1 immunoreactivity was attempted in *pdx1-cre*^Cre/0^; *Zeg*^GFP/0 ^e11.5 r1 tissue. No detectable signal was observed (Figure 7B), despite robust expression of GFP (Figure 7A) and reliable detection of Pdx1 in the adult pancreas under these conditions.

While this work was under review, two publications reported neuronal transgene expression in a variety of mouse lines used for the study of pancreatic development and function [58,59]. These results are consistent with and complementary to our results. Using the Pdx1-cre mouse line employed in our study, Wicksteed et al and Song et al report a similar pattern of Cre-mediated recombination in the developing and adult hypothalamus and brainstem. Furthermore, Wicksteed et al observed a similar pattern of recombination in an independently generated mouse line in which Cre expression is driven by a similar construct incorporating regulatory sequences upstream of the *pdx1 *gene, suggesting that the expression patterns we observe cannot be due solely to insertion site effects. They also report a lack of galactosidase activity in mice bearing a heterozygous LacZ insertion at the *pdx1 *locus and a lack of amplification of endogenous *pdx1 *transcript from the brain (both consistent with our observations, herein and unpublished). Taken together, their results indicate that *pdx1 *regulatory elements can drive highly specific neuronal expression, but that endogenous *pdx1 *is not expressed in the mouse brain, likely due to the activity of a repressor element not contained in the Pdx1-cre transgene constructs currently employed. The reported widespread expression of endogenous *pdx1 *reported in the rat brain may reflect a difference between gene regulation between rat and mouse or methodological differences between the rat and mouse studies [56,57].

## Conclusions

We report here, using the widely used Pdx1-cre line, a highly specific pattern of Cre-mediated recombination in the central nervous system and inner ear. This Cre line, and the regulatory sequences that direct Cre expression, may be a valuable resource for investigators seeking to manipulate gene expression in specific subsets of neurons, such as forebrain-projecting serotonergic neurons and neurons of the dorsomedial hypothalamus. To our knowledge, no other Cre mouse lines have been described to exhibit a pattern of hypothalamic recombination comparable to that we observed in the Pdx1-Cre line. However, the fact that recombination was observed in several hypothalamic nuclei which may have very different physiological functions (such as the arcuate and dorsomedial nuclei), as well as in the pancreas, may require further characterization of pdx1 regulatory elements in order to maximize the utility of *pdx1 *regulatory elements for this purpose.

At present, several transgenic methods have been developed which generate specific Cre-mediated recombination in serotonergic neurons [4,5,60]. Two of these methods involve the use of bacterial artificial chromosomes containing regulatory elements or genes expressed in serotonergic neurons, and unlike the Pdx1-cre line, result in recombination in the vast majority of serotonergic neurons [60,5]. However, like the Pdx1-cre line, these Cre transgenes may also result in recombination in other cell types, such as thalamocortical neurons [60] and pancreatic β-cells (Ohta, German et al, in submission). A technically sophisticated method has been described which allows for highly specific targeted recombination in subsets of serotonergic neurons; however, this method may be impractical for some investigators due to the complexity of genetic manipulations required [4].

It is important to note that the brain regions which exhibit recombination in the Pdx1-cre mouse line are well known to have important roles in *in vivo *metabolic function, including glucose homeostasis [61,53]. Investigators employing this line to express Cre in the developing pancreas should consider the possibility that concomitant Cre expression in the central nervous system may play a significant role in resulting *in vivo *phenotypes. Conversely, investigators who plan to use this mouse line to manipulate neuronal gene expression should consider the possible effects on pancreatic gene expression and function. These considerations are particularly salient when analyzing behavior and physiology in adult mice.

In conclusion, these data are consistent with the idea that common patterns of gene expression in pancreatic β-cells, serotonergic neurons and hypothalamic neurons contribute to their highly specialized and, in many cases, similar physiology.

## Competing interests

The authors declare that they have no competing interests.

## Authors' contributions

GH, MB, AL, MSG and LT conceived of experiments; GH and AL performed experiments; GH and AL analyzed data; GH wrote the manuscript; all authors edited the manuscript.
